# Gonadotrophin stimulation and risk of relapse in breast cancer

**DOI:** 10.1093/hropen/hoaa061

**Published:** 2021-01-16

**Authors:** A Fredriksson, E Rosenberg, Z Einbeigi, C Bergh, A Strandell

**Affiliations:** Department of Obstetrics and Gynecology, Sahlgrenska University Hospital, Gothenburg, SE 413 45, Sweden; Department of Obstetrics and Gynecology, Sahlgrenska University Hospital, Gothenburg, SE 413 45, Sweden; Department of Medicine, Southern Älvsborg Hospital, Borås, SE 501 82, Sweden; Department of Oncology, Southern Älvsborg Hospital, Borås, SE 501 82, Sweden; Institute of Clinical Sciences, Sahlgrenska Academy, University of Gothenburg, Gothenburg, SE 413 45, Sweden; Department of Obstetrics and Gynecology, Sahlgrenska University Hospital, Gothenburg, SE 413 45, Sweden; Institute of Clinical Sciences, Sahlgrenska Academy, University of Gothenburg, Gothenburg, SE 413 45, Sweden; Department of Obstetrics and Gynecology, Sahlgrenska University Hospital, Gothenburg, SE 413 45, Sweden; Institute of Clinical Sciences, Sahlgrenska Academy, University of Gothenburg, Gothenburg, SE 413 45, Sweden

**Keywords:** ovarian stimulation, breast cancer, relapse, IVF, fertility preservation, gonadotrophin, cryopreservation, pregnancy, live birth, survival

## Abstract

**STUDY QUESTION:**

Is gonadotrophin stimulation as part of IVF associated with an increased risk of relapse in breast cancer?

**SUMMARY ANSWER:**

Controlled ovarian stimulation (COS) in connection with IVF in women with previous breast cancer was not associated with an increased risk of breast cancer relapse.

**WHAT IS KNOWN ALREADY:**

Breast cancer is the most common malignancy among women worldwide and the leading cause of cancer death among females. The use of COS with gonadotrophins with subsequent cryopreservation of oocytes or embryos in order to enhance the chances of pregnancy after cancer treatment is the current most established fertility preservation method for women with breast cancer. To date, there are only a few small retrospective hospital-based controlled studies evaluating the risk of breast cancer relapse in patients undergoing fertility preservation with or without COS, showing no evident risk of relapse in breast cancer after the use of gonadotoxic agents.

**STUDY DESIGN, SIZE, DURATION:**

This was a retrospective, population-based cohort study comprising 5857 women with previous breast cancer of whom 337 were exposed to COS. Exposure (COS) and outcomes (relapse and death) were identified for all patients from 2005 to 2014 by assessing the National Quality Register for Assisted Reproduction, the Swedish Medical Birth Register, the National Patient Register, the Swedish Prescribed Drug Register, the Swedish Cause of Death Register, the National Breast Cancer Register and the Swedish Cancer Register. Matching according to set criteria was possible for 334 women, who constituted the control group. A total of 274 women had undergone IVF after completing breast cancer treatment and 63 women had undergone COS for fertility preservation at the time of breast cancer diagnosis.

**PARTICIPANTS/MATERIALS, SETTING, METHODS:**

Women aged 20–44 years previously diagnosed with breast cancer and exposed to COS were matched for age at breast cancer diagnosis ±5 years, tumour size and lymph node involvement with a non-exposed control group, including women with known T- and N-stages. In a subsequent analysis, the matched cohort was assessed by also including women with unknown T- and N-stages. A secondary analysis comprised the entire non-matched cohort, including all women with known T- and N-stages. Also here, a subsequent analysis included women with missing data for T- and N-stages. The risk of relapse in breast cancer was estimated as crude hazard ratios (HRs) and 95% CI using Cox proportional hazards models in the primary and secondary analyses where T- and N-stages were known: otherwise the risks of relapse were only given descriptively.

**MAIN RESULTS AND THE ROLE OF CHANCE:**

In the primary matched analysis, relapse occurred in 20 of 126 women exposed to COS (15.9%) compared with 39 of 126 (31.0%) in the control cohort (HR = 0.70; 95% CI 0.39–1.45; *P *=* *0.22). In the subsequent analysis, also including women with unknown T- and N-stages, relapse occurred in 27 of 337 (8.0%) women having undergone COS compared with 71/334 (21.3%) among the non-exposed. In the secondary adjusted analysis, relapse occurred in 20 of 126 (15.9%) exposed women and in 918 of 3729 (24.6%) non-exposed women (HR = 0.81; 95% CI 0.49–1.33; *P *=* *0.70). In the subsequent analysis, including unknown T- and N-stages, relapse occurred in 27 of 337 (8.0%) women in the exposed group and 1176 of 5520 (21.3%) in the non-exposed cohort.

**LIMITATIONS, REASONS FOR CAUTION:**

A substantial degree of missing data on important prognostic variables was a limitation, particularly when analysing the total cohort. Furthermore, data on confounding factors, such as BMI, were not completely covered. Another limitation was that a pre-specified variable for relapse was not in use for the majority of the National Breast Cancer Register. Furthermore, the follow-up time from available register data (2005–2014) is rather short. Finally, we cannot be sure whether the prognostic information from receptor status, showing a lower incidence in the exposed group, is representative. Information on T- and N-stages was missing in more than half of the patients.

**WIDER IMPLICATIONS OF THE FINDINGS:**

In this large, retrospective, matched cohort study, we found no increased risk of relapse in breast cancer among women who had been exposed to gonadotrophins as part of IVF. This is reassuring but might be confounded by the selection of a group of women with a more favourable prognosis than those not undergoing IVF. The present study strengthens previous findings by being large, national and register based. Its results are applicable to women undergoing fertility preservation as well as to those undergoing regular IVF treatment.

**STUDY FUNDING/COMPETING INTEREST(S):**

Supported in part by grants from the Swedish state under the agreement between the Swedish government and the county councils the ALF-agreement (ALFGBG-720291), The Assar Gabrielsson Fund (FB 15-20), The Breast Cancer Fund and the Swedish Association of Local authorities and Regions, SKR. There are no conflicts of interest to declare.

**TRIAL REGISTRATION:**

N/A

WHAT DOES THIS MEAN FOR PATIENTS?

For women who desire to have a child after breast cancer treatment difficulties in becoming pregnant may occur due to side effects of the chemotherapy used for treatment of the cancer. Some women may then use assisted reproduction techniques. The most common approach to preserving the ability to conceive after breast cancer is by hormonal stimulation of the ovaries with subsequent freezing of eggs (oocytes) or fertilised eggs (embryos) before cancer treatment begins. To date, there is limited knowledge showing no apparent risk for breast cancer relapse after hormonal stimulation of the ovaries. In the present study, women who had breast cancer and were treated with hormonal stimulation as part of IVF were compared with a group of women not exposed to such hormonal stimulation: we found that hormonal stimulation of the ovaries was not associated with an increased risk of relapse in breast cancer. This is a reassuring result. However, although many important risk factors were considered in our study, particularly the T- and N-stages as well as the receptor status, the group of women who underwent hormonal stimulation of the ovaries was probably a group with a better prognosis concerning breast cancer, compared with the women not undergoing ovarian stimulation.

## Introduction

Breast cancer is the most common malignancy among women in the world and the leading cause of cancer death among females, accounting for 23% of the total cancer cases and 14% of the cancer deaths (DeSantis *et al.*, 2014; Bray *et al.*, 2018). The 5-year age-standardized net survival has increased from 53% during the early 1970s to 87% in 2013 (Copson *et al.*, 2013). More recent data from NORDCAN describe a 5-year age standardised relative survival of 91–94% among women <50 years in the Nordic countries (NORDCAN, 2020). Although breast cancer represents ∼30% of all female cancer in Sweden, it is uncommon among women under 40 years of age, comprising only 4% of all cases (National Board of Health and Welfare, 2018). Nevertheless, young age has been shown to be an independent risk factor for a worse prognosis after breast cancer treatment (Fredriksson and Fredholm, 2017). Screening methods and advances in adjuvant therapies have contributed to the overall improved survival and decreased relapse rates (Siegel *et al.*, 2014).  

The treatment with gonadotoxic agents may affect ovarian function and result in subsequent subfertility, albeit the menstrual cycle often regularises after completing breast cancer treatment (Partridge *et al.*, 2010). As a consequence of this treatment, many women have raised concerns about their fertility and may seek ART, either as a means of fertility preservation (Guenther *et al.*, 2017) or treatment *per se* using controlled ovarian stimulation (COS) with gonadotrophins (Schover, 2005; Rosen *et al.*, 2009; Ruddy *et al.*, 2011; Benedict *et al.*, 2016).

Consequently, healthcare providers may raise questions regarding the risk of breast cancer and breast cancer relapse after both pregnancy and COS as they cause a systemic elevation of oestradiol. The association between oestrogen/progesterone levels and breast cancer is studied both in users of HRT and of oral contraceptives. The HABITS trial (Holmberg and Anderson, 2004), a randomised trial of administering HRT to women previously treated for breast cancer, was stopped prematurely owing to a higher frequency of relapse in breast cancer in the HRT group compared with placebo, indicating that elevated oestrogen/progesterone may be detrimental for these patients. The risk of relapse in breast cancer with oral contraceptive use has, to our knowledge, not been studied. A systematic review from Gierisch *et al.* (2013), including 44 studies, found a borderline increased risk of breast cancer in users compared to non-users (Odds ratio 1.08; 95% CI 1.00–1.17).

The general risk of breast cancer recurrence is highly dependent on stage, histology and tumour biology, and less on age (Nordenskjöld *et al.*, 2019).

Concerning any potential risk of pregnancy increasing the risk of relapse, several studies have shown that pregnancy after breast cancer treatment seems to be safe (Ives *et al.*, 2007; Kroman *et al.*, 2008; Azim *et al.*, 2011, 2013; Nye *et al.*, 2017; Lambertini *et al.*, 2018). A meta-analysis from Australia, including 1829 women, showed a lower risk of death in women with breast cancer and subsequent pregnancy compared with non-pregnant women (Hartman and Eslick, 2016). This finding has been confirmed by other studies (Iqbal *et al.*, 2017).

The risk of COS for developing breast cancer has been extensively studied. Two systematic reviews including eight and seven studies, respectively, and a large population-based register study suggested that IVF exposure does not raise the overall breast cancer risk (Sergentanis *et al.*, 2014; Gennari *et al.*, 2015; Lundberg *et al.*, 2017). A more recent large cohort study from the UK, including 255 786 women undergoing IVF, did not find any overall increased risk of invasive breast cancer while a small but significant increased risk was found for breast cancer *in situ* (Williams et al., 2018).

However, the research question of COS being associated with relapse in breast cancer has been sparsely investigated. A few retrospective hospital-based controlled studies have evaluated the risk of breast cancer relapse in patients undergoing fertility preservation with COS, with no evidence of increased risk of relapse in the COS cohort (Azim *et al.*, 2008; Kim *et al.*, 2016).

Our group has published a national population-based study assessing the association between birth after IVF and relapse in breast cancer compared with birth after natural conception, with no evidence of an increased risk (Rosenberg *et al.*, 2019), a result that was similar to a smaller study from Denmark (Goldrat *et al.*, 2015). In the present study, we included all COS cycles, not only those leading to pregnancy and live birth, thus aiming to investigate the association between COS and risk of relapse in breast cancer.

## Materials and methods

This is a retrospective, population-based, matched cohort study. We used national health and quality registries, which we cross-linked by unique personal identification numbers (PIN) to identify the population, exposure and outcome.

### Patients  

The National Patient Register (NPR), the National Quality Register for Breast Cancer and the Swedish Cancer Register were used to identify all women, aged 20–44 years, who were diagnosed with breast cancer from January 2005 to December 2014. These women formed the study base. The diagnosis malignant tumour in the female breast was coded as C50 in the International Classification of Diseases (ICD)10 (from 1997). We excluded all early-stage breast cancer defined as cancer *in situ* (Cis/Tis) and/or T0 according to the Tumour-, Node- and Metastasis staging system (TNM) as no evident risk for cancer relapse can be shown for these early-stage cancer categories (Moody-Ayers *et al.*, 2000).

### Exposure

Exposure to gonadotrophins was defined as either the start of a fresh IVF cycle including stimulation with gonadotrophins, as registered in the National Quality Register for Assisted Reproduction (Q-IVF) or the acquisition of a prescription of gonadotrophins from the Swedish Prescribed Drug Register. Between 2005 and the end of 2006, data were collected from the Swedish Prescribed Drug Register using the unique codes according to the Anatomical Therapeutic Chemical Classification System for all available prescribed gonadotrophins (code G03G). From 2007 and onwards, exposure data were collected from Q-IVF. We further identified the COS exposure subgroups for the purpose of fertility preservation by setting a time limit from breast cancer diagnosis to COS exposure to a maximum of 3 months. The main indication for IVF treatment was subfertility. This variable could not be individually assessed, since it was not registered in Q-IVF. The COS regimens included gonadotrophin stimulation in combination with a GnRH antagonist and in recent years an aromatase inhibitor from the start of the cycle.

### Non-exposure

Patients in the study base without any stimulated IVF-cycles or use of gonadotrophins formed the non-exposed cohort, from which controls were identified.

### Outcome 

The outcome was relapse in breast cancer. Relapse as a variable in the National Breast Cancer Register was reported only in one region in Sweden; Stockholm-Gotland. For this study, we created a coding template where we defined typical patterns of ICD diagnoses and procedures in the NPR that indicated relapse in breast cancer (Supplementary Table SI). This was carried out independently by two authors whose results were compared. Any disagreement was resolved in consensus with a third author with oncological expertise. The Stockholm-Gotland part of the National Breast Cancer Register was used as the true reference to which the defined relapse cases were compared.

The coding template was further elaborated by including and excluding diagnoses and treatments to achieve optimal sensitivity and specificity to correctly identify relapse before using it in the entire cohort.

### Variables  

Potential confounding factors affecting the risk of relapse in breast cancer were accounted for through available register data. The Medical Birth Register (MFR) provided data on previous childbirths and BMI at the start of pregnancy, as well as smoking (3 months before pregnancy). From the National Breast Cancer Register, information on tumour stage and hormone receptor status as well as a woman’s age at the time of diagnosis was collected. To define the time from diagnosis to exposure, we used the dates from the National Breast Cancer Register, the Swedish Cancer Register, the MFR, the Q-IVF and the Swedish Prescribed Drug Register.

### Data sources

The* Q-IVF* was established in 2007. The register covers ∼20 000 COS cycles annually, with treatment results and potential medical risks for both IVF children and their parents.

The* MFR*, which started in 1973 and covers almost all births in Sweden, has high validity (Cnattingius *et al.*, 1990). It includes information about maternal characteristics such as age, parity, height, weight, socioeconomic status and smoking habits.

The* NPR* includes all in- and outpatient care in Sweden comprising all main and secondary diagnoses as well as diagnostic and therapeutic procedures with dates of admission and discharge. Since 1987, all parts of Sweden have participated. Approximately 1% of cases are missing the main diagnosis.

The* Swedish Prescribed Drug Register* started in 2005 and is maintained by the National Board of Health and Welfare through the E-health Authority, to which all medical drugs sold are reported.

From the* Swedish Cause of Death Register*, also maintained by the National Board of Health and Welfare and with complete coverage since 1961, we received information on deaths caused by breast cancer in all Swedish citizens, both domestic and abroad.

The* National Breast Cancer Register* comprises six regional sections, each of which has started at different time points during the 1980s and with different coverage of specific variables. All primary breast cancer has been reported to a national register database (INCA) starting from 2008 (coded as C50) and categorised by gender, age, tumour stage (according to the TNM system) and procedures.

*The Swedish Cancer Register* was founded in 1958 and covers the entire population. Approximately 60 000 malignant cases of cancer are registered every year in Sweden.

### Statistical analyses

Descriptive statistics (using SAS System Version 9.4, SAS Institute, Cary, NC, USA) are presented with number and percentages for categorical variables and with mean, SD, median, minimum and maximum for continuous variables.

The primary analysis was conducted on the matched cohort in which each exposed woman was matched to one control and included women with known T- and N-stages. Matching variables were age at breast cancer diagnosis (±5 years), tumour size (T-stage at diagnosis; T1 (tumour <2 cm), T2 (<5 cm), T3 (>5 cm)) and lymph node involvement (N-stage 0 or 1). In a subsequent analysis, patients with unknown T- and N-stages were included.

A secondary analysis was conducted on the entire study base cohort, including patients with known T- and N-stages and with adjustment for these stages. A subsequent analysis with inclusion of patients without recorded T- and N-stages was also performed.

The risk of relapse in breast cancer was estimated as hazard ratios (HRs) using Cox proportional hazards models. We included each woman’s time at risk computed from the date of breast cancer until whichever event occurred first; relapse in breast cancer, death or end of the follow-up period. COS was included as a time-dependent variable, i.e. the time before ovarian stimulation was assigned as non-exposure and the time after COS as exposure. We estimated crude and adjusted HRs and 95% CIs. Significance level was set to 5%. In the analyses where T- and N-stages were unknown, the risks of relapse were only given descriptively.

### Ethical approval

The study has been approved by the Regional Ethical Committee at the University of Gothenburg (number 240-15). Informed consent was not required.

## Results

A total of 5857 women were included in the study base, of whom 337 were exposed to COS. Matching according to set criteria was possible for 334 women, who constituted the control group. A total of 274 women had undergone IVF after completed breast cancer treatment and 63 women (18.7%) had undergone COS for fertility preservation at the time of breast cancer diagnosis. The median time from the date of breast cancer diagnosis to the first COS exposure for those who underwent IVF after breast cancer treatment (n = 274) was 3.38 years ranging from 3 months to 12 years. Number of cycles per patient varied from one (30%) to nine, but only three patients underwent more than five cycles. Stimulation for fertility preservation was started within mean 49 days (range 6–89) from breast cancer diagnosis.

### Primary analysis

Background variables for women being exposed or not being exposed to gonadotrophins in the matched cohort with known T- and N-stages are presented in Table I. Among the non-matched variables, hormonal receptor-positive breast cancer (tested in a minority of patients) was less common among those exposed to gonadotrophins. In addition, previous childbirth was less common among exposed women. In the primary matched analysis, only including women with known T- and N-stages, relapse occurred in 20 of 126 women exposed to COS (15.9%) compared with 39 of 126 (31.0%) in the control cohort (HR = 0.70; 95% CI 0.39–1.45; *P *=* *0.22). Mean time from breast cancer diagnosis to relapse was 3.87 years (range 10 months to 10 years) in women exposed to COS and 3.50 years (6 months to 10 years) in non-exposed women.

**Table I:** Patient and tumour characteristics in the matched cohort, excluding women with unknown T- and N-stages, according to gonadotrophin exposure.

	Gonadotrophin exposure (n* *=* *126)	No gonadotrophin exposure (n* *=* *126)
Age at breast cancer diagnosis		
Years, mean (SD)	36.5 (5.5)	37.8 (4.9)
Median (min; max)	36.6 (20.2; 44.9)	38.6 (24.0; 45.0)
T (tumour), n (%)		
T1	74 (58.7)	74 (58.7)
T2	43 (34.1)	43 (34.1)
T3	8 (6.3)	8 (6.3)
T4	1 (0.8)	1 (0.8)
N (nodal), n (%)		
N0	89 (70.6)	89 (70.6)
N1	37 (29.4)	37 (29.4)
M (metastases), n (%)		
M0	104 (82.5)	110 (88.0)
M1	1 (0.8)	1 (0.8)
Missing	21 (16.7)	14 (11.2)
Oestrogen receptor, n (%)		
Positive	31 (24.6)	55 (43.7)
Negative	35 (27.8)	10 (7.9)
Missing	60 (47.6)	61 (48.4)
Progesterone receptor, n (%)		
Positive	27 (21.4)	45 (35.7)
Negative	39 (31.0)	19 (15.1)
Missing	60 (47.6)	62 (47.4)
HER2-sensitivity, n (%)		
Positive	8 (6.3)	12 (9.5)
Negative	56 (44.4)	52 (41.3)
Missing	62 (49.3)	62 (49,2)
Childbirth before breast cancer, n (%)	70 (55.6)	95 (75.4)
Smoking (3 months before pregnancy), n (%)	14 (25.5)	13 (19.7)
Missing	71 (53.7)	60 (47.6)
BMI (kg/m^2^)		
Mean (SD)	24.6 (4.2)	24.0 (4.2)
Median (min; max)	23.7 (17.0; 36.2)	23.1 (17.4; 41.2)
Missing, n (%)	55 (43.7)	45 (35.7)
Time to IVF (years)		
Mean (SD)	2.27 (2.60)	
Median (min; max)	1.47 (0.02; 10.85)	

For variables with missing values, numbers are given.

HER2, human epidermal growth factor receptor 2.

In the subsequent analysis, also including women with unknown T- and N-stages, relapse occurred in 27 of 337 (8.0%) women having undergone COS and in 71 of 334 (21.3%) among the non-exposed (Supplementary Table SII shows patient characteristics). Nine of 27 women with relapse of breast cancer died, corresponding to 2.7% among women exposed to COS. In the non-exposed group, 39 breast cancer-related deaths occurred (11.7%).

### Secondary analysis

In a secondary adjusted analysis, including only women with known T- and N- stages, relapse occurred in 20 of 126 (15.9%) exposed women and in 918 of 3729 (24.6%) non-exposed women (adjusted HR = 0.81; 95% CI 0.49–1.33; *P *=* *0.70). This analysis was adjusted for T- and N-stages (Table II).

**Table II:** Patient and tumour characteristics in the entire cohort, excluding women with unknown T- and N-stages, according to gonadotrophin exposure.

	Gonadotrophin exposure (n* *=* *126)	No gonadotrophin exposure (n* *=* *3729)
Age at breast cancer diagnosis		
Years, mean (SD)	36.5 (5.5)	40.2 (4.0)
Median (min; max)	36.6 (20.2; 44.9)	41.2 (21.6; 45.0)
T (tumour), n (%)		
T1	74 (58.7)	1948 (52.2)
T2	43 (34.1	1384 (37.1)
T3	8 (6.3)	323 (8.7)
T4	1 (0.8)	74 (2.0)
N (nodal), n (%)		
N0	89 (70.6)	2561 (68.7)
N1	37 (29.4)	1093 (29.3)
N2	0	60 (1.6)
N3	0	15 (0.4)
M (metastases), n (%)		
M0	104 (82.5)	3056 (82.2)
M1	1 (0.8)	64 (1.7)
Missing	21 (16.7)	597 (16.1)
Oestrogen receptor, n (%)		
Positive	31 (24.6)	1470 (39.4)
Negative	35 (27.8)	446 (12.0)
Missing	60 (47.6)	1813 (48.6)
Progesterone receptor, n (%)		
Positive	27 (21.4)	1297 (34.8)
Negative	39 (31.0)	613 (16.4)
Missing	60 (47.6)	1813 (48.8)
HER2-sensitivity, n (%)		
Positive	8 (6.3)	378 (10.1)
Negative	56 (44.4)	1491 (40.0)
Missing	62 (49.3)	267 (49.9)
Childbirth before breast cancer, n (%)	70 (55.6)	2918 (78.3)
Smoking (3 months before pregnancy), n (%)	14 (11.1)	262 (7.0)
Missing	71 (56.3)	2265 (60.7)
BMI kg/m^2^		
Mean (SD)	24.6 (4.2)	24.0 (3.9)
Median (min; max)	23.7 (17.0; 36.2)	23.3 (16.2; 46.0)
Missing, n (%)	55 (43.7)	1361 (36.5)

For variables with missing values, numbers are given.

HER2, human epidermal growth factor receptor 2.

A subsequent analysis included the entire cohort irrespective of known and unknown T- and N-stages, with 337 women exposed to COS and 5520 non-exposed women. In this cohort, the mean ages at diagnosis were 34.3 years and 40.0, respectively. Several predicting variables were disproportionally distributed between the two groups, including T- and N-stages (Supplementary Table SIII). In this analysis, relapse occurred in 27 of 337 (8.0%) women in the exposed group and in 1176 of 5520 (21.3%) in the non-exposed cohort.

### Outcome of coding

The coding template for relapse reached a 90% concordance with the Stockholm-Gotland part of the National Breast Cancer Register serving as the reference. The ability of the coding template to correctly identify relapses among ‘true’ relapses was 611/646 (0.95) and to correctly identify individuals without relapse was 1034/1183 (0.87), resulting in a sensitivity of 95% and specificity of 87%.

## Discussion

In this large, retrospective, matched cohort study, we evaluated the risk of relapse in breast cancer among women who had been exposed to gonadotrophins either at the time of breast cancer diagnosis or later, after completing breast cancer treatment, in comparison with those without any gonadotrophin exposure. By including both those who underwent fertility preservation and those who underwent a complete IVF treatment, we created a national complete cohort of women exposed to COS and increased the sample size, although the latter group had already been shown to have a reasonable prognosis by the time interval between breast cancer diagnosis and start of COS. Ovarian stimulation with gonadotrophins was not associated with an elevated risk for breast cancer relapse, neither in the primary matched analysis nor in the secondary analysis adjusted for T- and N-stages. These are reassuring results but are probably confounded by selection of a group of women with a more favourable prognosis than those who were not undergoing IVF. Comparing the non-exposed group with the COS exposed group, we noticed a 4-fold increase in breast cancer-related death (11.7% versus 2.7%), which may suggest a selection bias.

We tried to counteract selection bias by matching controls for age at breast cancer diagnosis as well as tumour size and lymph node involvement. From baseline data in the entire cohort, it can be assumed that women in the control group had a more advanced cancer with worse prognosis compared with those who have undergone COS, based on the more advanced T-stage and hormonal receptor sensitivity. Thus, only descriptive statistics are presented for comparisons including the entire cohort and no test for significance was conducted.

The evaluation of receptor data (oestrogen, progesterone and human epidermal growth factor receptor 2) was hampered by the poor reporting to the register, since these variables were not included from the start of the register: neither was the breast cancer gene (BRCA)-status part of the register. We cannot be sure whether the prognostic information from receptor status, showing a lower incidence in the exposed group, is representative. In addition, information on T- and N-stages was missing in more than half of the patients, since the register was not established until 2008.

There are few earlier reports studying the risk of breast cancer relapse after COS and overall, and there seems to be no support for a negative impact on the oncological prognosis. In an observational study from the USA, the relapse risk in 215 women exposed to COS combined with letrozole was compared with 136 women not undergoing COS. Although no difference in risk of recurrence was demonstrated ((3/79; 3.8% versus 11/136; 8.1%) (HR = 0.56; 95% CI 0.17–1.90)), the study was hampered by a small population (Azim *et al.*, 2008). In a systematic review, the largest study comprising 337 women reported a relapse rate of 5% (6/120) in the COS exposed cohort (with combined letrozole stimulation) compared with 5.5% (12/217) in the non-exposed control group (HR 0.77; 95% CI 0.28–2.13) (Rodgers *et al.*, 2017).

In a recent Swedish hospital-based matched cohort study comprising 148 women with previous breast cancer who underwent hormonal treatment for fertility preservation compared with a non-exposed control group, the incidence risk ratio (IRR) for relapse was 0.59 (95% CI 0.34–1.04) (Rodriguez-Wallberg *et al.*, 2018). After adjustments for T- and N-stages, chemotherapy and oestrogen receptor status, this age and calendar period adjusted outcome remained virtually unchanged (IRR 0.66; 95% CI 0.37–1.17). As the Swedish report further illustrated, the introduction of letrozole in 2010 may have had an advantageous impact on the risk for breast cancer relapse, as a potential proliferative effect on breast cancer cells due to both oestrogen and non-oestrogen modulated pathways is lowered by reduced systemic levels of oestrogen (Oktay *et al.*, 2010; Goldrat *et al.*, 2015; Rodgers *et al.*, 2017; Rodriguez-Wallberg *et al.*, 2018). Thus, letrozole may have a protective impact on the overall breast cancer survival in this subgroup of rather young women (Goldrat *et al.*, 2015). In addition, other hormonal influences, such as the effect of androgens, progestins and cytokines, still need to be studied further (Early Breast Cancer Trialists’ Collaborative Group, 1998).

### Strengths

The strengths of the present study are that data were collected on a national basis as opposed to previous hospital-based cohort studies, resulting in a large cohort, and that data from several registries were combined to control potential confounding. Using national registries gives a low risk of selection bias. By including patients from the time of breast cancer diagnosis until the completion of breast cancer treatment, the results are applicable to women undergoing fertility preservation as well as to those undergoing regular IVF treatment.

### Limitations

There are several limitations in this study. One is the substantial degree of missing data on several important prognostic variables, which may be explained by the recent start of both the Swedish Prescribed Drug Register in 2005 and the National Breast Cancer Register in 2008. Data on potential confounding factors, such as BMI and smoking, were not completely covered in the registries. Although demographic factors, such as high BMI and low parity, have been associated with an increased risk of breast cancer and the effect of smoking is unclear, less is known about their effect on relapse.

Another limitation is the lack of documented relapses. The coding template resulted in a sensitivity of 95% and a specificity of 87%, considered to be fairly good. It is, however, a limitation that a pre-specified variable for relapse was not in use in the register at that time. Furthermore, the follow-up time from available register data (2005–2014) is rather short.

## Conclusion

COS as a fertility preservation method or IVF treatment seems to be safe in women with previous breast cancer as no increased risk of relapse in breast cancer was observed in a matched cohort or when adjusted for N- and T-stages. This is reassuring but might be confounded by selection of a group of women with a more favourable prognosis than those not undergoing IVF.

## Supplementary data

Supplementary data are available at *Human Reproduction Open* online.

## Supplementary Material

hoaa061_Supplementary_Data
