# Supplementary material for: Gonadotrophin stimulation and risk of relapse in breast cancer
Source: Hum Reprod Open. 2021 Jan 16;2021(1):hoaa061. doi: 10.1093/hropen/hoaa061 (PMC7810817; doi:10.1093/hropen/hoaa061)
Supplement: hoaa061_Supplementary_Data [file hoaa061_supplementary_data.docx]

## Supplementary Table SI Code criteria for relapse (ICD10).

If any of the following occurs 1 year (365 days) (or 2 years (730 days) where indicated) or later from the first breast cancer diagnosis (C50):

1. Death caused by C50 C77 C78 C79 (first 3 positions) (after 2 years).
2. Visits with diagnosis C77 C78 C79 (first 3 positions) and at the same time C50 or Z853.
3. Visits with diagnosis C77 C78 C79 D05 (3 first positions) or Z510 Z511 and at the same time a diagnostic code for operation HAF.
4. Visit with diagnosis C77 C78 C79 D05 (first 3 positions) or Z510 Z511 and at the same time a diagnostic code for operation HAC but no code HAE at the same visit.
5. Visit with diagnosis C77 C78 C79 D05 or Z510 Z511 and at the same time a diagnostic code for operation PJA10 PJD42 PJD52.
6. Visit with diagnosis C77 C78 C79 D05 or Z510 Z511 and at the same time a code for operation TPX10.
7. Two or more occurrence of visits with diagnosis Z510 Z511 (after 2 years). Three or more occurrence of visits with diagnosis D051. Two or more occurrence of visits with diagnosis C77 C78 C79 D05 or Z510 Z511 and at the same time a code for operation DV069 DV070 DV071 DV072 DT107 DT108 DT112 DT116 DT135 (after 2 years).

C50 Malignant neoplasm of breast

C77 Secondary and unspecified malignant neoplasm of lymph nodes

C78 Secondary and unspecified malignant neoplasm of respiratory and digestive organs

C79 Secondary malignant neoplasm of other unspecified sites

Z510 Encounter of antineoplastic radiation therapy

Z511 Encounter of antineoplastic chemotherapy and immunotherapy

Z853 Personal history of malignant neoplasm of breast

D05 Lobular cancer in situ of breast, D051 Cancer in situ of breast cancer

HAF Local excision of cancer recurrence or metastasis

HAC Mastectomy, HAE Reconstruction of the breast

PJA10 Exploration of lymph nodes, PJD42 Extirpation of axillary lymph nodes, PJD52 radical extirpation of axillary lymph nodes

DV069 Radiation therapy unspecified, DV070 Intensity modified radiation therapy, DV071 External radiation therapy, DV072 Local radiation therapy

DT107 Chemotherapy, DT108 Administration of chemotherapy, DT112 Chemotherapy, intrathecal, DT116 Chemotherapy, intravenous administration, DT013 Intrathecal drug administration

TPX10 Application of intravascular catheter

**Supplementary Table SII** Patient and tumour characteristics in the matched cohort, including women with unknown T- and N-stages, according to gonadotrophin exposure.

|  | Gonadotrophin exposure (*n*=337) | No gonadotrophin exposure (*n*=334) |
| --- | --- | --- |
| Age at breast cancer diagnosis  Years, mean (SD)  median (min; max) | 34.3 (6.6) 35.0 (20.2; 44.9) | 35.8 (5.9) 36.6 (20.0; 45.0) |
| T (Tumour), n (%)  T1  T2  T3  T4  Missing, n | 75 (57.7)  45 (34.6)  8 (6.2)  2 (1.5)  *n*=130  207 | 75 (56.8)  45 (34.6)  8 (6.2)  2 (1.5)  *n*=130  204 |
| N (Nodal), n (%)  N0  N1  Missing, n | 89 (70.6)  37 (29.4)  *n*=126  211 | 89 (70.6)  37 (29.4)  *n*=126  208 |
| M (Metastases), n (%)  M0  M1  Missing, n | 104 (98.1)  2 (1.9)  *n*=106  231 | 113 (97.4)  3 (2.6)  *n*=116  218 |
| Oestrogen receptor, n (%)  Positive  Negative  Missing, n | 32 (47.8)  35 (52.2)  *n*=67  270 | 57 (82.6)  12 (17.4)  *n*=69  265 |
| Progesterone receptor, n (%)  Positive  Negative  Missing, n | 28 (41.8)  39 (58.2)  *n*=67  270 | 47 (69.1)  21 (30.9)  *n*=68  266 |
| HER2-sensitivity, n (%)  Positive  Negative  Missing, n | 8 (12.5)  56 (87.5)  *n*=64  273 | 12 (17.9)  55 (82.1)  *n*=67  267 |
| Childbirth before breast cancer, n (%) | 177 (52.5) | 223 (66.8) |
| Smoking, n (%)  (3 months before pregnancy)  Missing, n | 37 (23.7)  *n*=156  181 | 37 (23.7)  *n*=156  178 |
| BMI kg/m^2^  mean (SD)  median (min; max)  Missing, n | 24.3 (4.0) 23.1 (17.0; 36.7) *n*=195  142 | 24.4 (4.3) 23.5 (16.7; 41.2) *n*=193  141 |

HER2= human epidermal growth factor receptor 2. For variables with missing values, numbers are given.

**Supplementary Table SIII** Patient and tumour characteristics in the entire cohort, including women with unknown T- and N-stages, according to gonadotrophin exposure.

|  | Gonadotrophin exposure (*n*=337) | No gonadotrophin exposure (*n*=5520) |
| --- | --- | --- |
| Age at breast cancer diagnosis  Years, mean (SD)  median (min; max) | 34.3 (6.6) 35.0 (20.2; 44.9) | 40.0 (4.2) 41.1 (20.0; 45.0) |
| T (Tumour), n (%)  T1  T2  T3  T4  Missing, n | 75 (57.7)  45 (34.6)  8 (6.2)  2 (1.5)  *n*=130  207 | 1991 (52.2)  1408 (37.0)  333 (8.7)  79 (2.1)  *n*=3811  1709 |
| N (Nodal), n (%)  N0  N1  N2  N3  Missing, n | 89 (70.6)  37 (29.4)  0 (0)  0 (0)  *n*=126  211 | 2586 (68.6)  1107 (29.4)  60 (1.6)  15 (0.4)  *n*=3768  1752 |
| M (Metastases), n (%)  M0  M1  Missing, n | 104 (98.1)  2 (1.9)  *n*=106  231 | 3117 (97.5)  79 (2.5)  *n*=3196  2324 |
| Oestrogen receptor, n (%)  Positive  Negative  Missing, n | 32 (47.8)  35 (52.2)  *n*=67  270 | 1521 (76.4)  471 (23.6)  *n*=1992  3528 |
| Progesterone receptor, n (%)  Positive  Negative  Missing, n | 28 (41.8)  39 (58.2)  *n*=67  270 | 1348 (67.8)  639 (32.2)  *n*=1987  3533 |
| HER2-sensitivity, n (%)  Positive  Negative  Missing, n | 8 (12.5)  56 (87.5)  *n*=64  273 | 398 (20.5)  1543 (79.5)  *n*=1941  3579 |
| Childbirth before breast cancer, n (%) | 177 (52.5) | 4204 (76.2) |
| Smoking, n (%)  (3 months before pregnancy)  Missing, n | 37 (23.7)  *n*=156  181 | 394 (18.9)  *n*=2081  3439 |
| BMI kg/m^2^  mean (SD)  median (min; max)  Missing, n | 24.3 (4.0) 23.1 (17.0; 36.7) *n*=195  142 | 24.1 (4.0) 23.3 (13.0; 59.2) *n*=3404  2116 |

For variables with missing values, numbers are given.
